# COVID-19 and Systemic Racism Pandemics Impact Daily Life for Immigrant/Refugees, Minneapolis, USA

**DOI:** 10.5334/aogh.3411

**Published:** 2021-11-09

**Authors:** Mary O. Hearst, Angela Ekwonye, Leso Munala, Halimo Ismail, Erika Kennedy, Heather Buesseler

**Affiliations:** 1 St. Catherine University, US; 2 Hennepin County, US

## Abstract

**Background:**

The pandemics of COVID-19 and systemic racism have a deleterious impact on the daily life experiences and health for populations of color. The experiences are compounded for immigrant/refugee communities that may have other barriers such as English language literacy or trauma. Cumulative stress due to everyday racism is harmful for health.

**Objectives:**

This study describes the impact of day-to-day lived experiences of Karen, Somali, and Latinx communities during the COVID-19 pandemic and aftermath of the police murder of George Floyd in the Minneapolis/St. Paul metro area.

**Methods:**

In-depth interviews were conducted over three weeks in September and October 2020 to understand the daily life experiences of Karen, Somali and Latinx adults drawn from community contacts during the COVID-19 pandemic and the aftermath of the police murder of George Floyd. Interviewers were bilingual and from the communities they interviewed. Nine questions were asked, ranging from their knowledge of COVID-19, prevention practices, experiences during shelter-in-place, and the perceptions of the police murder of George Floyd. Qualitative analysis included transcript review, coding facilitated by Atlas.ti Cloud software, summaries, and validation by interviewers.

**Findings:**

Thirty-two adults were interviewed (Latinx = 10, Karen = 10, Somali = 12). One-third were in person per participant request and complying with COVID-19 precautions, and the remainder were remote. The average age recorded was 37 years (range 20–66 years), 43.8% males and 56.3% females. Respondents reported experiences of discrimination and systemic racism while engaging in daily life activities, including accessing foods and common goods, school, work, transportation, and healthcare, all of which were exacerbated by COVID-19 and the police murder of George Floyd.

**Conclusions:**

Immigrant/refugee communities of color in Minneapolis/St. Paul face daily experiences of racism that were compounded by the events of 2020. Discrimination and systemic racism contribute to the persistent health inequities among populations of color.

## Background

The U.S. is in the midst of a centuries-old pandemic of racism that contributes to health inequities for Black, Indigenous, and Persons of Color (BIPOC), including immigrant/refugee communities [1]. Persistent and daily experiences of racism contribute to higher rates of morbidity and mortality [2] with substantial mental health consequences [3]. BIPOC communities experience elevated and prolonged levels of stress while engaging in daily life activities due to experiences of racism, trauma, and negative thinking [4]. This increases cortisol levels, which contribute to higher rates of morbidity and mortality [5,6].

Social and structural inequities for immigrants/refugees in the U.S. have also long existed, including institutional racism in health care [7]. The Minneapolis/St. Paul metro area hosts the largest communities of Karen [8] (the most recent Asian immigrant population from Burma/Myanmar) and Somali immigrants and refugees in the U.S., and Minnesota’s largest immigrant population is Latinx [9]. The Latinx community is largely Mexican heritage (70%), followed by Puerto Rican, El Salvadorian, and Guatemalan [10]. The Karen community began immigrating to Minnesota in the early 2000, coming from oppressive Burmese government and refugee camps. The Karen refugees face language barriers, low literacy in both their primary language and English, and challenges adapting to transportation, extremely cold weather and other technology, from using an oven to a computer [11]. Xenophobia, microaggressions, and racism are persistent for Asian (“China-virus”) [12,13], Latinx (undocumented) [14], and Somali (duality of Black and Muslim) immigrants/refugees [15]. The current experiences of xenophobia and a lack of access to healthcare resources among undocumented immigrants [14,16,17] perpetuates health inequities.

The COVID-19 pandemic has amplified both awareness of the racialized systems and differential impact that immigrant/refugee populations of color experience. The inequities among BIPOC communities has been consistent since the beginning of the pandemic in early 2020 and contribute to the higher impact of COVID-19 on these groups [14,18,19,20,21]. Current data sources provide little information by sub-population [22]; however, as of September 16, 2021, the Minnesota age-adjusted incidence rates per 100,000 population of COVID-19 infection were 8,368 among Asians; 13,061 among Black, and 19,228 among Latinx, compared to 9,529 among whites [23]. The proportion of cases among Black and Latinx communities was higher than their proportion of the total population; Asians were similar, and whites were lower [23]. This is consistent with what is occurring nationally, with the exception that Asian communities have fewer cases but higher than expected hospitalizations and deaths [24].

Particular to immigrant/refugee communities, a number of factors present differential risk and lead to poorer health outcomes and higher morbidity and mortality rates [13,14,20,21,25,26]. As first generation immigrants, health care access and social safety nets are not well-established, particularly for those who are undocumented [14,27,28]. Information about COVID-19 is not uniformly accessible, and trusted sources are not universally available in these communities [14,27,29,30]. Limited English proficiency reduces access to information [20], coupled with limited parental ability to assist their children with remote learning [26,31]. For illustration, sixty percent of Karen adult refugees in the U.S. have limited English proficiency [22,32]. Many immigrant/refugee communities live in multigenerational homes and are more likely to hold essential worker roles [33], placing themselves and their family members at greater risk of COVID-19 [13,21,25,26].

The police murder of George Floyd on May 29, 2020 launched nationwide protests, the most fervent of which occurred in Minneapolis and St. Paul, Minnesota. Businesses and property were burned down and looted in the same neighborhoods at highest risk of COVID-19 [34]—neighborhoods with high concentrations of immigrants/refugees. Curfews were in place throughout Minneapolis and St. Paul to quell the riots. Extensive damage was done in both Minneapolis and St. Paul; however, the most extreme was in the heart of a low-income, immigrant community, resulting in damage to 1300 properties and $3.5 million in damages [35]. Remarkably, the protests did not cause a surge in COVID-19 cases [36]; however, existing mental health problems and social isolation accelerated, notably among Black and Asian adults [37]. Further compounding this stress was the closure of places of worship and limitations on social gatherings, which serve as critical centers for community information and psychosocial support for successful integration [38].

The purpose of this study is to describe the lived daily experiences of Somali, Karen and Latinx adults in the social and political context of 2020. It is imperative to understand how the immigrant/refugee communities’ experiences of the co-occurrence of COVID-19 pandemic and the aftermath of a police murder of George Floyd affect the daily life experiences of these communities in order to develop policies during and after the pandemic that mitigate—not exacerbate—pre-existing health inequities and social vulnerabilities.

## Methods

### Design

This study is an exploratory, qualitative study to understand the daily lived experiences of members of immigrant/refugee communities during the co-occurrence of the COVID-19 pandemic and the aftermath of police murder of George Floyd. It is rooted in a contextual constructionist framework [39]. A constructionist epistemology is based on the notion of permeability. It suggests that truth is not objective but is constructed through the interaction of research participants and the interviewers [40]. This perspective maintains that results will vary according to how the data was collected and analyzed (context). Context in this study includes investigators’ and participants’ cultural and personal histories, including the observations’ immediate setting [39].

### Participants

Purposive and snowball sampling were used to recruit adults 18 years or older from the Karen, Latinx, and Somali refugee and immigrant groups in the Minneapolis/St. Paul metro area. Participants had to have gone through the resettlement process to the U.S.; if U.S.-born to immigrant parents, they were excluded from the study. Recruitment aimed for variation in age, gender identification, socioeconomic status, and profession. Only one member per household was eligible.

### Data collection

Data were collected using in-depth interviews (IDIs). Interviewers were recruited based on their interest in the research and representativeness of the Karen, Somali and Latinx community including proficiency in English and Karen, Somali and Spanish. Interviewers were trained in conducting IDIs over two sessions, practiced with a trusted source, and debriefed with the Principal Investigators for any revisions to the questions. Interviews occurred in September and October 2020. Interviews were conducted over the phone via video-conferencing software, or in person, practicing COVID-19 social distancing guidelines. Interviews were recorded with permission using a digital device and were transcribed. Interviews not conducted in English were translated from Karen, Spanish or Somali to English for analysis. Each interview lasted 30 to 60 minutes. Informed consent was obtained from each participant who was offered a $50 gift card for completing the interview. The St. Catherine University Institutional Review Board approved this research (Protocol #1442).

### Measures

The interviews included nine questions, although this manuscript focused on findings drawn primarily from two questions. The questions were 1) “In the past 3–6 months, we have all been experiencing shelter-in-place and transitioning to more open movement. Has the pandemic or the ‘shelter-in-place’ orders brought about additional strain with anyone in your household or elevated stress?” Prompts included concerns of safety, employment or loss of income, children at home, access to computers, language proficiency to help children with school work, and food access; and 2) “In addition to the COVID pandemic, the nation and the Twin Cities has had a rise in social unrest, police violence and destruction of property. In what ways has the aftermath of police violence, murder of George Floyd, impacted you?” Prompts included stress, afraid to leave the house, change in access to groceries, loss of businesses, and racial discrimination. Participant age, sex and ethnicity were the only demographics asked.

### Data Analysis

Qualitative data analysis software (Atlas.ti Cloud) was used to code and analyze the transcripts. During data analysis, the team first read through all transcripts, built and refined a coding scheme, coded the transcripts, reconstructed the data into themes and patterns, interpreted the results, and checked findings with the interviewers. A disaggregated analysis by immigrant/refugee sub-group was done to ascertain unique experiences of each community group. The findings are a representation of many possible truths and experiences articulated by participants. From the research team’s perspective, knowledge and reality are socially constructed, so the research findings are one of many possible interpretations of how participants are making sense of the COVID-19 pandemic, George Floyd’s death, and the aftermath. With a focus on impact and experiences due to COVID-19 and the aftermath of police murder George Floyd’s death, each daily life activity was analyzed based on unique and targeted experiences of racism related to COVID-19, systemic racism, or both.

## Results

A total of 32 adult participants were interviewed for this analysis (Latinx = 10, Karen = 10, Somali = 12). One-third of the interviews (n = 8) were conducted in-person and the remainder were conducted remotely. The average age recorded was 37 years (range 20–66 years). There were 14 (43.8%) males and 18 (56.3%) females interviewed.

Daily life activities included in this analysis were 1) accessing food and essential goods; 2) school; 3) work; 4) transportation; and 5) accessing healthcare.

**Access to food and essential goods**: Participants described the difficulties they experienced getting food and other essential goods, as well as the experience of discrimination and the fear of contracting COVID-19 while shopping for these items. Participants reported a rise in food prices and the subsequent consequence that federal nutrition assistance (food stamps) no longer stretches as far as it used to. Participants reported some difficulties, particularly early in the COVID-19 outbreak, in accessing necessary food and supplies because stores were out of supplies or limiting quantities that could be purchased. For example, one Karen participant described, “So we usually go to Sunfood, but they ran out of rice. The next place we went to was Sam’s Club to get rice, but they also ran out of rice.” While this was a common occurrence, the addition of the social unrest following George Floyd’s death destroyed businesses that for many immigrants/refugees was their only accessible source of food and essential goods. A Somali participant reported, “I have lots of friends that couldn’t find places to get food when the stores got shutdown. Thus, I had to take it upon myself to buy them food and go to stores for them.” A Latinx participant shared, “[There are] no stores anymore. I cannot go to the grocery store, I don’t have a pharmacy close, our bank, everything is gone.”

Multiple participants reported fear of shopping for food and essential goods. Several described fear of contracting COVID-19 while out in the community and bringing it home to their family, like this Latinx participant: “[It] is stressful to live with the fear that you can get infected. You need to be away from people all the time. They cannot touch anything without you maybe getting infected. You can’t go to the store …because all is closed or restricted. [There are] no places to enjoy—just live with fear.” Other participants, particularly Karen, described fear related to the xenophobic environment that stemmed from COVID-19 being dubbed the “China virus.” Karen participants reported being targeted for and experiencing discrimination in the community for causing the pandemic. This Karen participant explained:

There was this time where I was paying for my groceries, and I was trying to pay; this cashier was trying to get far away from me. They acted like they were afraid of me and like they were looking down on me. I don’t really understand it myself, because they were acting like they were trying to protect themselves. They probably thought, “This Asian person has the disease,” and they were trying to rush me; I paid for my things and left.

Still others described fear related to the social unrest following George Floyd’s death. In some cases, participants explained the compounding effects of the social unrest, COVID-19, and systemic racism impacting their access to food and essential goods.

Because of this we’re having to stay at home, not having much food, having low income, even [not] receiving information. So, this all comes together. So even with COVID or riots/destructions that has happened, it affects me and my family.” [Somali]

Those just become a thought, but it does stop my learning and me from going out. It also stops us from getting food and also started economic hardship, like our family money. It’s really scary for us. [Karen]

I can tell you many times where they stopped me at the stores or anywhere and try to verify if I’m buying something, who am I, who was there. They ask me for my driver’s license all the time. [Latinx]

**School**: Participants discussed school from various perspectives: participants who are students, a member of a household with siblings who attends school, or a parent of children attending school. The daily challenges of school specific to the Karen, Somali and Latinx communities were linked to access to necessary materials, language and education, and support services. Not all families had access to the necessary materials for themselves or their child’s online education, including computer equipment that includes a camera and adequate internet access. One Latinx parent stated the need for support in the following way, “At school, Latino kids need more support…[schools need] to send the kids home with a laptop [with] Wi-Fi. Latino kids need more tutors because their parents have no education.”

The most significant challenge to home-based remote learning was the parent’s English language learning status and their education level.

The biggest problem is that we came from Asia and refugee camps, so we don’t know how to speak English. The laws and rules, we don’t understand anything. Since we came to America, things like computers, iPad or passwords, I don’t know anything about it. So now that they have online learning, I don’t know what to do. I go to school to pick [up] my child’s iPad and book, but when I come back home, I don’t know how to use the iPad. Things like what the teachers are saying and sending, I don’t understand anything, so this is a concern for me. [Karen]

[Much] of the homework was very difficult to understand…I have to talked to older women [who] don’t speak English [and] are in need to help their kids for school, and they don’t know what to do—they are very stressed out. Not many women have the education to use the technology to start with, even if they have some education—that’s not the same…we are in America, for example, the history class it’s not the same at the history class we took in Mexico. [Latinx]

In some cases, the inability for the parents to help their children in school adds more family obligations to older siblings. As this Karen parent described, “Her older brother can help her, but if he doesn’t know how to help her, then there is no one who can help her with her homework. I can’t help her with her homework because I don’t know the language.” [Karen]

**Work/Vocation**: Participants commonly reported substantial financial challenges due to COVID-related under- and unemployment. Participants described being laid off, having their hours reduced, and needing to be at home because of home schooling. These hardships along with increased household costs resulted in financial stress among many participants.

Bills are higher because of the stay-at-home or being in the house more, using more electricity. And it’s hard to pay those bills when you can’t work and when you need the money that you’re getting for food for the children. How are we going to pay those bills if we cannot feed the children? [Latinx]

I have been working for 19 years, and we have been told to just go home because of the pandemic, and that we have closed our workplace so just go home. [Somali]

The social unrest following George Floyd’s death and curfew during and in the days after these events added to participants’ financial struggles. This Karen participant described, “When there is a curfew, I don’t get to work the full shift and [have to] return home. I am scared of the virus, plus the curfew. I get tardy and earn less money due to the curfew.” Others described trepidation going to and from work safely, as well as fear that the violent unrest would seriously destabilize their families’ lives (a fate they had already survived with the events that precipitated their resettlement):

I think it was the second day of the riot when it hit Saint Paul. […] And then the buildings that burned down, it was very close to my workplace, so we were very scared […] that [our] building is going to be burned down. Because if it’s burned down, then I would lose my job. If I lose my job, how am I going to pay for the rent, and then if I/we can’t pay for the rent, then I might get kicked out of the house and [we] will become homeless, you know? [Karen]

The damage in the aftermath of the social unrest did cause additional financial hardship among some participants. One participant clearly described this trauma and the implications for Latinx and Somali business owners.

I do not agree with all the situation to break and burn stores. Most of the stores they damaged were family stores for Latino people or Somali people who live on Lake Street, and unfortunately the only people who was damaged was these families who have been working really hard to have their business, and now they’re gone. I don’t know I agree with the people who come and destroy the stores and steal and burn and break everything. That’s no point. That’s no real protest. We want to change the system, but that is not the right way, and it’s not fair, and the only people who really was more affected was the people of color itself—the Latino people the Somali people and the people who basically are poor. [Latinx]

Even greater financial burdens were described among undocumented Latinx participants. One person described, “I’m so distressed because we lost our job, and we don’t, I don’t have the same income, and that affects me. I don’t qualify for a stimulus checks because of my immigration status.”

One Latinx participant specifically mentioned that her financial vulnerability made her more vulnerable to virus transmission, because she does not have the type of job where she can work from home or afford things like grocery delivery. In her words:

I think that as a Latina, it makes me more vulnerable, because for example, my supervisor and her husband can afford to work from home, they can afford to buy the groceries online and being delivery, and they can afford to pay the extra $10 charge for the delivery. It’s something that I cannot do, because I need to $10 dollars to pay stuff. So I guess people with low income are more vulnerable to be exposed [to COVID-19].

Many participants described working in essential roles—healthcare workers, teachers, delivery jobs, retail, etc. Yet, many participants expressed the most concern about transmitting COVID-19 to family members. This Somali participant noted, “The biggest issue I was faced with was the pandemic, and just fear of bringing COVID to my kids after working, or them bringing it home causing me to spread it to those I work with.”

Furthermore, despite statewide worker-protection mandates, participants described that they were applied inconsistently. One Somali participant described the positive measures their workplace was implementing:

Yes, so I work at the [redacted]. There is a lot of problems. However, at my work, since the beginning of the pandemic, they have set strict rules, such as weekly tests, as well as you can’t enter the building without wearing a mask. Additionally, your temperature will also be checked to see if you have a fever. Therefore, then I hear some of the workers that are sent home, be told that they have a fever or if they test positive their told to stay home for two weeks.

However, a Karen participant reported a negative experience:

I am also scared of the virus, but my workplace said we could not stay home. Many of the workers asked to stay home and for the workplace to shut down, but they refused to shut the workplace down, not even one day. Even though we are scared, we have to go to work. If we don’t go, we will get tardy. We also don’t understand the law, and we can’t speak English. We also don’t understand the company rules, so I go to work every day.

Despite many stresses associated with working during the pandemic and events after George Floyd’s death, participants also found new meaning in their work in the midst of these hardships. This Somali participant expressed deep meaning and purpose in her role in healthcare: “And then going into the health care field and just finding myself, finding my voice, finding how I can, you know, even step up from not only myself but for my patients, you know, and not be demeaned for being a black Muslim woman that’s bilingual in the healthcare field.” A Karen participant expressed renewed passion and purpose in her recent degree:

I think when it comes to, in terms of health, we talk about George Floyd, we talk about police brutality, I think for me, I am personally really passionate about that, I just graduated from social worker department, so definitely [care about] policies, social justice, humanity, health, all these issues […] are very important for me. That’s why I mentioned that talking about hard issues and rude issues of racism, police brutality is very important.

**Transportation**: Participants noted transportation challenges that exacerbated the above-reported daily life challenges, including food/supplies shortages, racially targeted interactions, and generalized fear of being stopped by the police escalated by George Floyd’s death. The COVID-19 food shortage and reduced hours required participants to travel farther to access goods, as noted by this Karen participant: “My car is broken down. It’s hard for me, because the store that is close is closed, so I have to go to a store that’s far, and when I come home [from work], the store is already closed. My car is already broken, so if I go further, it’s going to break more.”

Participants also described generalized and accelerated fear of police racial violence, including traffic altercations. One Karen participant expressed, “Personally, I never get pulled over or stopped by the police, so I don’t know how to say it… But sometimes I’m worried about what would happen if police ever stop me. What are they going to do to me if they pull me over, because I am Asian, I am not white race?”

Use of public transportation also made participants vulnerable to racist interactions. This Latinx participant describes, “Well it has affected me a lot. I mean, [I am] definitely harassed by racist comments— like I go to take a bus, and people in the bus tell me that they voted for president [Trump] so they can deport me.”

**Healthcare experience**: Participants’ healthcare experiences can be categorized into access and experiences/perceptions. From an access perspective, many participants knew where to access healthcare and find a COVID-19 test. Many participants were receiving care for chronic conditions and, in general, those were being managed, albeit differently. In describing healthcare access for a family member, a Karen participant stated, “Well, she’s been going to like the doctor less than before the pandemic. She would often go like a few times a week, but now it’s like once like every other week. They also have a nurse coming in to check on her though. So, I guess that’s pretty good.”

Despite access to care, participants shared the experience of health disparities from a personal perspective, related to family members and societal implications. The health disparities participants experienced were explicit, related to pre-existing conditions, equitable access to care and equitable access to information. One example is the consequences Asians experience due to COVID-19. One Karen participant describes it as,

Yes, I have faced racial discrimination as an Asian or as a Karen person. Whenever I go, I’m scared that they might look down on me. They might think that I’m lonely or I smell bad. I’m really scared when I go to the hospital and I think that they might make fun of me and also might be unfair to me because I’m Asian.

In other cases, participants reported fears and concerns about the hospitals themselves and what happens at the hospitals. A Somali participant said, “I have worries because so many of my relatives and Somalis have contracted the virus, and when they were taken to the hospitals, very few of them came back from the hospital. They all died, and that itself made me have so much fear.”

Many participants described discrimination as an important cause of health disparities. Discrimination occurs at the system level and with individual healthcare professional interactions. As with other aspects of daily living, added racial stress due to police violence is compounded onto negative experiences with healthcare. One Somali participant described it as, “Also if you look at from COVID-19 perspective, with all that has happened with policy brutality, with COVID-19 and how some African American patients are treated, [they] are way worse because of medical racism.” Another example of the intersectionality of these issues is described by another Somali participant in this way,

[The healthcare systems are not helping] the minority community, you know, on how to [get] better employment, [have] better procedures. [The healthcare system] is not dealing with healthcare discrimination, which is very evident, sadly enough. So that’s basically what we’ve been dealing with. And it’s very hard to see that, because you’re already dealing with the whole pandemic situation and then also dealing with the disparity side of it. It took a toll.

## Discussion

COVID-19 is having profound economic and health impacts across the U.S. Mortality, unemployment, food insecurity and housing instability all have been elevated with persistent inequities among BIPOC communities [41]. This paper presents the additive impacts and increased vulnerabilities that immigrants/refugees face as BIPOC because of who they are and where they live and work. More specifically, this paper aims to present how COVID-19 and the concurrent police murder of George Floyd and related social unrest impacted daily activities among Somali, Karen, and Latinx immigrants/refugees in Minneapolis/St. Paul. The findings illuminate and validate published quantitative literature and news reports about the long-standing inequities faced by immigrants and refugees [15,16,17,20,28,30,42,43].

In this research population, participants experienced heightened fear of COVID-19 and systemic racism while engaging in daily activities. Consistent with trends nationwide, the pandemic brought with it increased economic insecurity and difficulties accessing food. Compared to non-Hispanic whites, Blacks and Hispanic adults had greater increase in food insecurity between 2018–2020 [44], greater cuts in consumption due to loss of employment (50% higher cut for Blacks and 20% higher for Hispanic) [45], and lower percentages of people with enough liquid assets to cover two-months of rent [46]. Financial stress only added to participants’ risk of COVID transmission, as they are not able to avail themselves of certain prevention modalities (e.g., grocery delivery) or may have to carpool or take public transit. Jobs common among this population increase potential exposure, as many work in essential roles or feel unable to push back on poor workplace prevention policies for fear of termination. Homeschooling was challenging for all immigrant/refugee parents who face language and technology barriers and lack familiarity with the U.S. educational system.

Stress from struggling with daily life during the pandemic is compounded by co-occurring experiences of microaggression and systemic racism, whether at the grocery store, doctor’s office, or on public transit. This is consistent with experiences among BIPOC across the U.S., with 39 percent of Asian, 38 percent of Black, and 27 percent of Hispanic adults reporting adverse experiences due to their race or ethnicity since the pandemic began. Karen participants are doubly discriminated not only as a person of color, but also due to accusations they brought COVID to the U.S. Nationwide, violence and discrimination against Asian Americans during COVID has surged [47,48]—hate crimes against this population in 16 major cities rose by 150 percent in 2020 [49]. This follows a persistent and well-documented trend [50,51] whereby immigrants are associated with germs and contagion and subsequently blamed and stigmatized for epidemics (HIV, TB, cholera, and now COVID-19), despite evidence [52] that immigrants are generally healthier than host populations. Anti-immigrant rhetoric has a history of politicizing disease, and policies are developed that systemically excludes immigrants from accessing healthcare [50] and other public services. The cumulative effect deters many immigrants from seeking medical care, giving rise to negative health consequences [50], and ultimately undermining health equity [53].

Immigrant/refugee communities may be particularly triggered by witnessing police violence and/or experiencing racial discrimination due to the traumatic events that precipitated their resettlement [54]. The Karen are a minority ethnic group originally from Myanmar (Burma) who were terrorized by Burmese soldiers. Their villages were burned, civilians tortured and killed, and women and girls raped, forcing entire communities to relocate internally or flee across the border to Thailand [55]. Somalis fled their country starting in 1991 when the state collapse and devolved into civil war. Since then, persistent conflict, drought, flooding, food shortages, and sectarian violence have caused hundreds of thousands to seek asylum or refugee status [56]. Latinx immigrants in the U.S., particularly those from the northern triangle of Central American (El Salvador, Guatemala, Honduras, and Mexico) have left their home countries due to gang violence, political persecution, human trafficking, and extreme poverty—a context that has been compared to war zones [57,58,59].

Previous trauma coupled with discrimination due to COVID-19 [60], increasing anti-immigrant rhetoric, and systemic racism may exacerbate health impacts among immigrants and refugees. The literature shows that experiences of discrimination among immigrants, refugees, and asylees results in significantly worse mental and physical health [1,61,62,63]. Racial discrimination is associated with clinical indicators of disease [64], cardiovascular disease [65], and risk factors [66], and health behaviors [67,68]. Research on stress and health includes, but is not limited to a combination of the stress of the experience as well as the constant state of hypervigilance when navigating situations in which a person may anticipate discrimination [64]. Chronically elevated levels of cortisol, a result of stress and/or hypervigilance and a “dysregulated hypothalamic-pituitary-adrenal axis, appear to mediate effects of racial discrimination on allostatic load and disease.” [6] Hence, cumulative effects of stress and trauma, to which immigrants/refugees are more susceptible due to their historical experiences, biologically link systemic racism and health inequities for BIPOC communities [13]. Furthermore, collective trauma is intergenerational and cumulative [69,70]. These effects are magnified in populations, like those under consideration in this study, that have been historically persecuted, marginalized, or have experienced the types of extreme trauma that caused them to flee their home country. This suggests a pressing and urgent need to interrupt these cycles among all BIPOC, especially among immigrants/refugees, or risk worsening health outcomes and inequities in the future.

Addressing the upstream causes of inequities for immigrant/refugee populations requires a multifaceted approach. First, it is vital to engage community leaders in the distribution of information and be included in the decision-making spaces, even more so during community such as the COVID-19 pandemic and response and police violence [25]. Immigrant/refugee communities benefit from cultural brokers, community health workers and other trained and trusted community-level supports for information and access [71,72]. At the institution level, institutional policies and practices are necessary to mitigate the experiences of xenophobia and healthcare microaggressions [29] that limit seeking care and the quality of care experienced. For example, the use of interpreters in the healthcare environment for immigrant/refugees is crucial for both language and culture; however, interpreters are not necessarily acknowledged and considered a key member of the healthcare team which limits their potential effectiveness [73]. Another key institution is the education system. Not all immigrant/refugee populations benefit from the ‘immigrant advantage’ [74] leaving many immigrant/refugee children behind. The education system requires adequate support to meet the needs of the students, including the necessary tools for effective home-based learning due to COVID-19. Data are important for understanding the distribution and etiology of health and disease, but much of the reported data do not present the heterogeneity among population groups [18,75,76]. There needs to be better and more documentation, especially for subpopulations given the heterogeneity within BIPOC populations to assure equity in access and services [12,27,77].

The final example is of political will. The officer who murdered George Floyd was convicted on April 20, 2021 – the first white officer charged due to the death of a black man [78]. In addition, the U.S. Justice Department is initiating an investigation of excessive force systematically used by Minneapolis Police Department. While this accountability is the first of many steps, the validation of the wrongdoing shows political will for systemic change that will benefit all BIPOC communities, including the fear and mistrust highlighted by the immigrant/refugees in this study.

This study has several limitations. The sample size, sample selection method and qualitative methodology limit generalizability of these findings. Unique interviewers were used for each population group to represent each culture and language; therefore, the interview quality may have varied, resulting in a different depth of content among groups. The quality of interview transcription and translation also varied by language group, which may affect the comparability of disaggregated analysis. Due to COVID-19 adaptation, the interviews were conducted on the phone, video conference via computer or in-person using prevention practices. It is possible that this variation may have altered the participants full engagement in interviews by mode. Finally, limited demographic data was collected to respect confidentiality thus limiting understanding any variance due to education or economic-status.

## Conclusion

Immigrants/refugees are experiencing significant hardship on daily life during the co-occurrence of COVID-19 and the aftermath of George Floyd’s death. They are more vulnerable than other BIPOC groups due to specific vulnerabilities as immigrants & refugees (eg., language barriers, lack of familiarity with navigating U.S. systems, inability to support their children in distance learning, lack of work options, documentation status). The added stress makes them even more susceptible to long-term mental and physical health impacts, suggesting the need for targeted public health interventions and social/economic policy and support mechanisms.

## References

[1] Williams DR, Lawrence JA, Davis BA. Racism and health: Evidence and needed research. Annu Rev Public Health. 2019; 40: 105–125. DOI: 10.1146/annurev-publhealth-040218-043750 30601726 PMC6532402

[2] Simons RL, Lei MK, Klopack E, Zhang Y, Gibbons FX, Beach SRH. Racial discrimination, inflammation, and chronic illness among African American women at midlife: Support for the weathering perspective. J Racial Ethn Health Disparities; 2020. DOI: 10.1007/s40615-020-00786-8 PMC8183614 32488825

[3] Sanchez M, Diez S, Fava NM, et al. Immigration stress among recent Latino immigrants: The protective role of social support and religious social capital. Soc Work Public Health. 2019; 34(4): 279–292. DOI: 10.1080/19371918.2019.1606749 31033427 PMC9872174

[4] Somerville K, Neal-Barnett A, Stadulis R, Manns-James L, Stevens-Robinson D. Hair cortisol concentration and perceived chronic stress in low-income urban pregnant and postpartum black women. J Racial Ethn Health Disparities; 2020. DOI: 10.1007/s40615-020-00809-4 32613440

[5] Williams DR. Stress and the mental health of populations of color: Advancing our understanding of race-related stressors. J Health Soc Behav. 2018; 59(4): 466–485. DOI: 10.1177/0022146518814251 30484715 PMC6532404

[6] Berger M, Sarnyai Z. “More than skin deep”: Stress neurobiology and mental health consequences of racial discrimination. Stress. 2015; 18(1): 1–10. DOI: 10.3109/10253890.2014.989204 25407297

[7] Feagin J, Bennefield Z. Systemic racism and U.S. health care. Soc Sci Med. 2014; 103: 7–14. DOI: 10.1016/j.socscimed.2013.09.006 24507906

[8] Culture Care Connection. Karen People in Minnesota. http://www.culturecareconnection.org/matters/diversity/karen.html. Published 2021. Updated 2020. Accessed April 5, 2021.

[9] American Immigration Council. Fact Sheet: Immigrants in Minnesota. Accessed August 6, 2020.

[10] Kolnick J. Minnesotanos: Latino Journeys in Minnesota. MNOPEDIA. https://www.mnopedia.org/minnesotanos-latino-journeys-minnesota. Published 2016. Accessed September 21, 2021.

[11] Karen Organization of Minnesota. Karen History. https://www.mnkaren.org/history-culture/karen-history/. Published 2017. Accessed September 21, 2021.

[12] Ho CP, Chong A, Narayan A, et al. Mitigating Asian American bias and xenophobia in response to the coronavirus pandemic: How you can be an upstander. J Am Coll Radiol. 2020; 17(12): 1692–1694. DOI: 10.1016/j.jacr.2020.09.030 33035504 PMC7538066

[13] Zhang M, Gurung A, Anglewicz P, Yun K. COVID-19 and immigrant essential workers: Bhutanese and Burmese refugees in the United States. Public Health Rep. 2021; 136(1): 117–123. DOI: 10.1177/0033354920971720 33207130 PMC7856376

[14] Macias Gil R, Marcelin JR, Zuniga-Blanco B, Marquez C, Mathew T, Piggott DA. COVID-19 pandemic: Disparate health impact on the Hispanic/Latinx population in the United States. J Infect Dis. 2020; 222(10): 1592–1595. DOI: 10.1093/infdis/jiaa474 32729903 PMC7454709

[15] Abdulle H. “Time to uproot systemic racism”: Case of Dolal Idd highlights disparities in fatal police shootings. Sahan Journal. January 5, 2021; 2021.

[16] Okonkwo NE, Aguwa UT, Jang M, et al. COVID-19 and the US response: Accelerating health inequities. BMJ Evid Based Med; 2020. DOI: 10.1136/bmjebm-2020-111426 PMC7299650 32493833

[17] Page KR, Venkataramani M, Beyrer C, Polk S. Undocumented U.S. Immigrants and Covid-19. N Engl J Med. 2020; 382(21): e62. DOI: 10.1056/NEJMp2005953 32220207

[18] Patton S. The pathology of American racism is making the pathology of the coronavirus worse. https://www.washingtonpost.com/outlook/2020/04/11/coronavirus-black-america-racism. Published April 11, 2020.

[19] Jones C. Confronting institutional racism. Phython. 2002; 50: 7–22. DOI: 10.2307/4149999

[20] Greenaway C, Hargreaves S, Barkati S, et al. COVID-19: Exposing and addressing health disparities among ethnic minorities and migrants. J Travel Med. 2020; 27(7). DOI: 10.1093/jtm/taaa113 PMC7454797 32706375

[21] King D. Coronavirus has been especially dangerous to immigrants. Here’s why. The Columbus Dispatch. November 28, 2020; 2020.

[22] Le TK, Cha L, Han HR, Tseng W. Anti-Asian xenophobia and Asian American COVID-19 disparities. Am J Public Health. 2020; 110(9): 1371–1373. DOI: 10.2105/AJPH.2020.305846 32783714 PMC7427240

[23] Minnesota Department of Health. Minnesota COVID-19 response data by race/ethnicity. https://mn.gov/covid19/data/data-by-race-ethnicity/index.jsp. Published September 16, 2021. Accessed September 21, 2021.

[24] Centers for Disease Control and Prevention. COVID-19 hospitalization and death by race/ethnicity. https://www.cdc.gov/coronavirus/2019-ncov/covid-data/investigations-discovery/hospitalization-death-by-race-ethnicity.html. Published Nov 30, 2020. Accessed February 9, 2021.

[25] Nguyen LH, Drew DA, Graham MS, et al. Risk of COVID-19 among front-line health-care workers and the general community: A prospective cohort study. The Lancet Public Health. 2020; 5(9): e475–e483. 32745512 10.1016/S2468-2667(20)30164-X PMC7491202

[26] Cholera R, Falusi OO, Linton JM. Sheltering in place in a xenophobic climate: COVID-19 and children in immigrant families. Pediatrics. 2020; 146(1). DOI: 10.1542/peds.2020-1094 32345687

[27] Behbahani S, Smith CA, Carvalho M, Warren CJ, Gregory M, Silva NA. Vulnerable immigrant populations in the New York metropolitan area and COVID-19: Lessons learned in the epicenter of the crisis. Acad Med. 2020; 95(12): 1827–1830. DOI: 10.1097/ACM.0000000000003518 32452838 PMC7268828

[28] Wilson FA, Stimpson JP. US policies increase vulnerability of immigrant communities to the COVID-19 pandemic. Ann Glob Health. 2020; 86(1): 57. DOI: 10.5334/aogh.2897 PMC7292131 32566486

[29] Acharya KP, Subramanya SH, Neupane D. Emerging pandemics: Lesson for one-health approach. Vet Med Sci; 2020. DOI: 10.1002/vms3.361 PMC7675301 33030806

[30] Peters J. Minnesota’s communities of color are suffering the most from COVID-19. Clinics that serve them are trying to get vaccins in arms-fast. Sahan Journal. February 4, 2021; 2021.

[31] Rani RS. Imagine online school in a language you don’t understand. New York Times. April 22, 2020; 2020.

[32] Lopez G, Ruiz N, Patten E. Key facts about Asican American, a diverse and growing populations. Pew Research Center September 8, 2017.

[33] Orrenius PM, Zavodny M. Do immigrants work in riskier jobs? Demography. 2009; 46(3): 535–551. DOI: 10.1353/dem.0.0064 19771943 PMC2831347

[34] Penrod J, Sinner CJ, Webser MH. Buildings damages in Minneapolis, St. Paul after riots. July 13, 2020; 2020.

[35] Bakst B. Owners of burned, looted businesses plead for state help. MPR2021.

[36] Dave DM, Friedson AI, Matsuzawa K, Sabia JJ, Safford S. Black Lives Matter protests and risk avoidance: The case of civil unrest during a pandemic. National Bureau of Economic Research Working Paper Series; 2020. No. 27408. DOI: 10.3386/w27408

[37] Fowers A, Wan W. Depression and anxiety spiked among black Americans after George Floyd’s death. The Washington Post. June 12, 2020; 2020.

[38] Agyekum B, Newbold BK. Religion/spirituality, therapeutic landscape and immigrant mental well-being amongst African immigrants to Canada. Mental Health, Religion & Culture. 2016; 19(7): 674–685. DOI: 10.1080/13674676.2016.1225292

[39] Stiles WB. Quality control in qualitative research. Clinical Psychology Review. 1993; 13(6): 593–618. DOI: 10.1016/0272-7358(93)90048-Q

[40] Madill A, Jordan A, Shirley C. Objectivity and reliability in qualitative analysis: Realist, contextualist and radical constructionist epistemologies. Br J Psychol. 2000; 91(Pt 1): 1–20. DOI: 10.1348/000712600161646 10717768

[41] Bauer L, Broady KW, O’Donnell J. Ten facts about COVID-19 and the U.S. economy. Brookings Institute; 2021.

[42] Tirupathi R, Muradova V, Shekhar R, Salim SA, Al-Tawfiq JA, Palabindala V. COVID-19 disparity among racial and ethnic minorities in the US: A cross sectional analysis. Travel Med Infect Dis. 2020; 38: 101904. DOI: 10.1016/j.tmaid.2020.101904 33137491 PMC7603979

[43] Fielding-Miller RK, Sundaram ME, Brouwer K. Social determinants of COVID-19 mortality at the county level. PLoS One. 2020; 15(10): e0240151. DOI: 10.1371/journal.pone.0240151 33052932 PMC7556498

[44] Schanzenbach D, Pitts A. How much has food insecurity risen? Evidence from the census household pulse survey. Evanston, IL: Institute for Policy Research Northwestern University; 2020.

[45] Ganong P, Jones D, Noewl P, Greig F, Farrell D, Wheat C. Wealth, race and consumption smoothing of typical income shocks. Cambridge, MA: National Bureau of Economic Research; 2020. DOI: 10.3386/w27552

[46] Kolomatsky M. How has the pandemic affected rent and mortgage payments? New York Times. June 18, 2020.

[47] Ruiz N, Horowitz J, Tamir C. Many black and Asian Americans say they have experienced discrimination amid the COVID-19 outbreak. www.pewresearch.org. Published 2020. Accessed 2021.

[48] Nuyen S. Anti-Asian attacks rise during pandemic. NPR2021; Race.

[49] Center for the Study of Hate and Extremism. Anti-Asian hate crime reported to police in America’s largest cities: 2019 & 2020. CSUSB; 2021.

[50] Markel H, Stern A. The foreignness of germs: The persistent association of imigrant and disease in American society. Milbank Quarterly. 2002; 80(4): 757–788. DOI: 10.1111/1468-0009.00030 PMC2690128 12532646

[51] Farmer P. AIDS and accusation: Haiti and the geography of blame. Berkeley, CA: University of California Press; 2006. DOI: 10.1525/9780520933026

[52] Abubakar I, Devakumar D, Madise N, et al. UCL-Lancet Commission on Migration and Health. Lancet. 2016; 388(10050): 1141–1142. DOI: 10.1016/S0140-6736(16)31581-1 27650082

[53] Holmes S. Fresh fruit, broken bodies: Migrant farmworkers in the United States. Berkeley: University California Press; 2013.

[54] Hameed S, Sadiq A, Din AU. The increased vulnerability of refugee population to mental health disorders. Kans J Med. 2018; 11(1): 1–12. DOI: 10.17161/kjm.v11i1.8680 PMC5834240 29844851

[55] Human Rights Watch. “They came and destroyed our village again”: The plight of internally displaced persons in Karen State. https://www.hrw.org/report/2005/06/09/they-came-and-destroyed-our-village-again/plight-internally-displaced-persons. Published 2005. Accessed April 21, 2021.

[56] Hammond L. History, overview, trends and issues in major Somali refugee displacements in the near region. Policy Development and Evaluation Service: UNHCR; 2014.

[57] UNHCR. Regional response to the Northern Triangle of Central America Situation; 2016.

[58] Palencia G. Poverty, violence drive Central American exodus. Reuters. June 30, 2014.

[59] Medecins Sans Frontieres. Force to Flee Central America’s Northern Triangle: A neglected Humanitarian Crisis; 2017.

[60] Zhang M, Gurung A, Anglewicz P, Baniya K, Yun K. Discrimination and stress among Asian refugee populations during the COVID-19 pandemic: Evidence from Bhutanese and Burmese refugees in the USA. Journal of Racial and Ethnic Health Disparities; 2021. DOI: 10.1007/s40615-021-00992-y PMC7924016 33651371

[61] Ziersch A, Due C, Walsh M. Discrimination: A health hazard for people from refugee and asylum-seeking backgrounds resettled in Australia. BMC Public Health. 2020; 20(1): 108. DOI: 10.1186/s12889-019-8068-3 31992261 PMC6986068

[62] Szaflarski M, Bauldry S. The effects of perceived discrimination on immigrant and refugee physical and mental health. Adv Med Sociol. 2019; 19: 173–204. DOI: 10.1108/S1057-629020190000019009 31178652 PMC6553658

[63] Ellis BH, MacDonald HZ, Lincoln AK, Cabral HJ. Mental health of Somali adolescent refugees: The role of trauma, stress, and perceived discrimination. J Consult Clin Psychol. 2008; 76(2): 184–193. DOI: 10.1037/0022-006X.76.2.184 18377116

[64] Lewis TT, Cogburn CD, Williams DR. Self-reported experiences of discrimination and health: Scientific advances, ongoing controversies, and emerging issues. Annu Rev Clin Psychol. 2015; 11: 407–440. DOI: 10.1146/annurev-clinpsy-032814-112728 25581238 PMC5555118

[65] Lewis TT, Williams DR, Tamene M, Clark CR. Self-reported experiences of discrimination and cardiovascular disease. Curr Cardiovasc Risk Rep. 2014; 8(1): 365. DOI: 10.1007/s12170-013-0365-2 24729825 PMC3980947

[66] Bernardo CO, Bastos JL, Gonzalez-Chica DA, Peres MA, Paradies YC. Interpersonal discrimination and markers of adiposity in longitudinal studies: a systematic review. Obes Rev. 2017; 18(9): 1040–1049. DOI: 10.1111/obr.12564 28569010

[67] Gilbert PA, Zemore SE. Discrimination and drinking: A systematic review of the evidence. Soc Sci Med. 2016; 161: 178–194. DOI: 10.1016/j.socscimed.2016.06.009 27315370 PMC4921286

[68] Slopen N, Lewis TT, Williams DR. Discrimination and sleep: A systematic review. Sleep Med. 2016; 18: 88–95. DOI: 10.1016/j.sleep.2015.01.012 25770043 PMC4699868

[69] De Gruy J. Post-traumatic slave syndrome. Uptone Press; 2005.

[70] Sotero M. A conceptual model of historical trauma: Implications for public health practice and research (Fall 2006). Journal of Health Disparities Research and Practice. 2006; 1(1): 93–108.

[71] Divine M. ‘Cultural broker’ is working to keep Karen community safe from COVID-19. Pioneer Press. October 18, 2020; 2020.

[72] Dobrzycka A. Applying the community health worker model to the immigrant and refugee population in Pittsburgh, PA: Public Health, University of Pittsburgh; 2008.

[73] Brisset C, Leanza Y, Laforest K. Working with interpreters in health care: A systematic review and meta-ethnography of qualitative studies. Patient Educ Couns. 2013; 91(2): 131–140. DOI: 10.1016/j.pec.2012.11.008 23246426

[74] Crosnoe R, Turley RNL. K-12 educational outcomes of immigrant youth. The Future of Children. 2011; 21(1): 129–152. DOI: 10.1353/foc.2011.0008 21465858 PMC5555844

[75] Lerner S. Coronavirus Numbers Reflect New York City’s Deep Economic Divide. The Intercept Web site. http://www.theintercept.com. Published April 9, 2020.

[76] APM Research Lab. The Color of Coronovirus. The APM Research Lab. https://apmresearchlab.org/covid/deaths-by-race. Published April 10, 2020.

[77] Coelho CM, Suttiwan P, Arato N, Zsido AN. On the nature of fear and anxiety triggered by COVID-19. Front Psychol. 2020; 11: 581314. DOI: 10.3389/fpsyg.2020.581314 33240172 PMC7680724

[78] Hayes M, Macaya M, Mahtani M, Wagner M, Rocha V, Berlinger J. Derk Chauvin guilty in death of George Floyd. CNN. April 20, 2021.

